# Transforming global water cycle observations via synergistic AI and remote sensing

**DOI:** 10.1126/sciadv.aef3610

**Published:** 2026-06-17

**Authors:** Zhaoyuan Yao, Yaokui Cui, Zhenong Jin, Shengbiao Wu, Qingyu Guo, Yue Xu, Zhizhou Guo, Chenchen Jiang, Yifan Qu, Dechao Zhai, Wenjie Fan

**Affiliations:** ^1^ Institute of Remote Sensing and Geographic Information System, School of Earth and Space Sciences, Peking University, Beijing, China.; ^2^ Beijing Key Laboratory of Spatio-temporal Perception and Urban Resilience, Beijing, China.; ^3^ Institute of Ecology, College of Urban and Environmental Science, Peking University, Beijing China.; ^4^ State Key Laboratory for Vegetation Structure, Function and Construction (VegLab), Peking University, Beijing, China.; ^5^ Future Urbanity and Sustainable Environment (FUSE) Lab, Division of Landscape Architecture, Department of Architecture, Faculty of Architecture, The University of Hong Kong, Hong Kong, China.; ^6^ Key Laboratory of Microelectronic Devices and Circuits (MoE), School of Integrated Circuits, Peking University, Beijing, China.; ^7^ College of Information Technology, Shanghai Ocean University, Shanghai, China.

## Abstract

Rapid shifts in terrestrial water cycle and water-related disasters due to climate change challenge the capability of remote sensing observation. Current models that rely on region-specific empirical parameters and multistage workflows fail to robustly handle complex and accelerated water cycle with high spatiotemporal resolutions. Increasing parameter complexity for fine-scale representation under various terrestrial conditions becomes prohibitive. To address these limitations, we present bidirectional encoder representations from transformers for hydrology (BERTH), an end-to-end artificial intelligence (AI) framework for global water cycle monitoring at 30-meter and daily scale, directly transforming remote sensing radiance to evapotranspiration, precipitation, soil moisture, and runoff. As a transformative paradigm for quantitative Earth science, BERTH trained on global datasets precisely captures spatiotemporal dynamics across diverse conditions, surpassing existing geophysical models in accuracy. Embodying the synergistic integration of AI and remote sensing, BERTH offers a transformative platform that supports next-generation investigations into global water resources and Earth system dynamics.

## Teaser

High-resolution artificial intelligence unifies global water cycle monitoring at daily scale to track Earth’s hydrological pulse.

## INTRODUCTION

The terrestrial water cycle, governing the movement and transformation of water across Earth’s spheres, fundamentally shapes human societies and regional economic trajectories (*1*–*3*). Accurately quantifying its key components—evapotranspiration (ET), precipitation (P), soil moisture (SM), and runoff (RO)—is critical for understanding Earth system dynamics and advancing the harmonious codevelopment of society and nature (*4*, *5*). However, dual pressures from climate change and anthropogenic forcing are accelerating the hydroclimate whiplash, coupled with frequent and abrupt disasters, including droughts, floods, and wildfires (*6*–*11*). The escalating spatiotemporal volatility of the Earth’s water cycle demands an urgent paradigm shift of water cycle monitoring, toward prioritizing high-resolution intelligence to mitigate water-related risks in an increasingly volatile world (*12*).

Over the past two decades, rapid advancements in Earth observation technologies, particularly remote sensing, have revolutionized global water cycle research, expanding its scope and precision (*13*–*16*). This precise remote sensing quantification of these water cycle variables now provides an indispensable tool for safeguarding climate stability, balancing ecosystems, and underpinning the sustainable development of human societies (*17*–*20*). Traditionally, methods for retrieving water cycle variables have predominantly relied on physical models driven by remote sensing data (*2*, *21*). While these models provide valuable mechanistic insights and underpin the first generation of global water cycle datasets, they face pivotal constraints (*1*, *2*, *22*). Specifically, these physical models typically focus on isolating individual variables, overlooking critical couplings within the water cycle (*23*). Furthermore, conventional approaches typically consist of a sequence of independent steps, including retrieval of biophysical parameters, spatial downscaling and temporal reconstruction, and calibration of empirical parameters. Their application to remote sensing data with diverse spatial resolutions, across large areas of heterogeneous land cover and complex terrain, introduces substantial challenges. These stem from their multistage estimation workflows and limited tunable parameters, which lead to cumulative error propagation and substantial cascading uncertainties (*24*–*26*). Therefore, datasets derived from these models often lack the requisite spatiotemporal granularity to meet the demands of research and applications in water cycle dynamics, global change, food security, and related fields.

The integration of artificial intelligence (AI) with remote sensing heralds an emerging paradigm for Earth science (*27*, *28*). Powered by big data and advanced computing, AI has achieved transformative success and underscored the potential of data-driven approaches (*29*–*31*). AI-based models leverage end-to-end design to extract complex patterns from big Earth data, offering a compelling solution to overcome the limitations of classical physical models and fundamentally reshaping remote sensing and Earth system science (*32*–*36*). Although Earth foundation models have revolutionized remote sensing applications, they remain insufficient for quantitatively retrieving high-resolution water cycle variables. These models are primarily optimized for coarse-scale atmospheric prediction or static land cover classification, rather than the complex processes of the terrestrial water cycle. Such frameworks lack the specialized mechanisms to capture the rapid, high-resolution spatiotemporal dynamics essential for characterizing global water cycle. Bridging these critical gaps in global water cycle monitoring through the synergy of AI and remote sensing offers an unprecedented opportunity in providing the precision required to mitigate global water-related risks in a rapidly changing climate.

To address these fundamental challenges, we propose the bidirectional encoder representations from transformers for hydrology (BERTH), a large-scale end-to-end quantitative remote sensing model. Built upon the convergence of big data, advances in physical models, and capabilities of data-driven AI, BERTH uses a holistic framework to directly transform remotely sensed radiance into high-resolution global water cycle variables. It extracts high-fidelity water cycle information by learning physical model outputs and ground-truth observations, rather than designing complex empirical parameterization schemes (*37*–*40*). BERTH’s exceptional adaptability delivers robust water cycle estimates across diverse land cover types and complex terrains, outperforming conventional physical models. These advancements position BERTH as a transformative framework in water cycle modeling. By quantitatively retrieving high-resolution water cycle variables, the model offers unprecedented potential to advance fundamental Earth system science, inform evidence-based water governance policies, and enable precision management of water resources.

## RESULTS

### Transformer-based architectures driven by remote sensing enable water cycle modeling

**Fig. 1.**
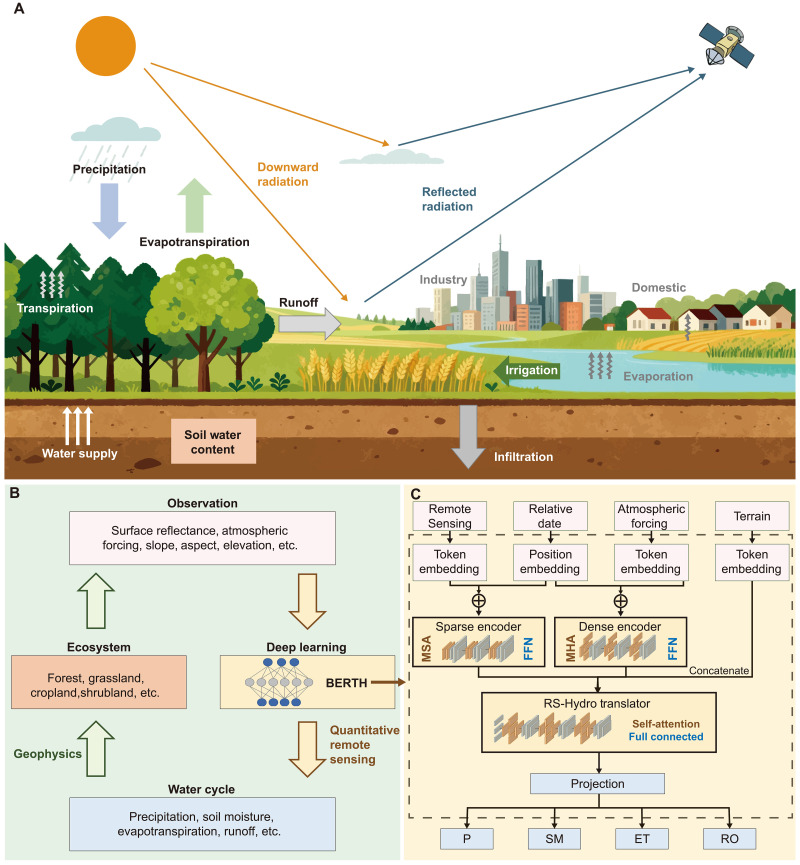
Overview of the BERTH pipeline and framework. (**A**) Mechanisms of hydrological remote sensing. (**B**) BERTH is considered as an efficient model in quantifying water cycle using remote sensing. (**C**) Detailed architecture of the BERTH model. MHA, multihead self-attention; MSA, masked self-attention; FFN, feed-forward network; RS-Hydro translator, remote sensing hydrological translator.

In this section, we conceptualize the architecture of the BERTH framework. The development of the BERTH model is predicated on the fundamental understanding that the hydrological conditions are inherently determining ecosystems and coupled to the surface radiation budget, allowing for remote sensing monitoring of surface hydrology (Fig. 1A). The terrestrial system, driven by downward radiation and P, partitions water inputs into ET, RO, infiltration, and changes in soil water content. Long-term P conditions determine the types of terrestrial ecosystems, whereas short-term moisture conditions affect the growth states of vegetation. Then, the reflected radiation observed by satellites is governed by surface properties (e.g., albedo, canopy structures, and surface moisture status). Recognizing that remote sensing provides only discrete samplings of land surface, we developed BERTH to retrieve continuous spatiotemporal hydrological patterns, effectively bridging the gaps between fragmented observations (Fig. 1B).

BERTH uses an advanced deep learning framework based on the transformer architecture, designed to map multimodal inputs onto key hydrological state variables (Fig. 1C). BERTH contains three modules: an embedding module that handles various input data, a large translator module containing three transformer encoders that decode hydrological information from input data, and an output module that extracts the hydrological space from the hidden space. (i) For the embedding module, the sequence of remote sensing observations and atmospheric drivers are processed as time-series tokens, enhanced by position embeddings to retain temporal order. (ii) The translator module takes advantage of several types of attention. High-resolution remote sensing data are extremely sparse across spatiotemporal scales, and filling them with default values introduces notable artifacts. Therefore, the sparse encoder and dense encoder are designed to extract features from discrete remote sensing and continuous atmospheric forcing, respectively. The masked self-attention (MSA) layers guarantee that continuous time series can be recovered from discrete remote sensing observations, whereas the multihead self-attention layers allow the model to dynamically weigh the importance of past and present observations. Remote sensing hydrological translator consists of stacked transformer encoder layers, which effectively learn the nonlinear multiscale dependencies between surface radiation and water cycle variables. (iii) The hidden representation is passed through a projection layer in the output module to simultaneously predict the target water cycle variables: ET, P, SM, and RO. Traditional transformers require filling in the complete sequence with default values to handle multisource inputs, which introduces notable artifacts and compromises attention fidelity at high spatiotemporal resolutions. BERTH’s improved architecture is capable to effectively translate the high-frequency, multispectral information captured by satellite instruments into temporally continuous and physically consistent estimates of water cycle components, overcoming the limitations imposed by discrete observational sampling.

### The BERTH model outperforms other advanced global geophysical models

**Fig. 2.**
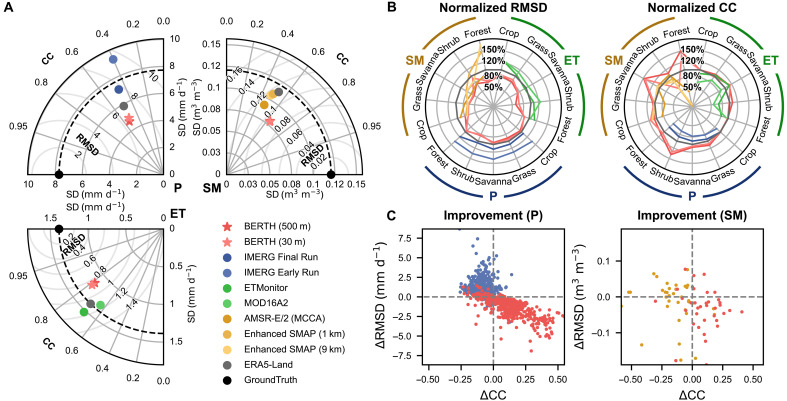
Model performance evaluation based on ground-truth data. (**A**) Overall performances of BERTH and other global datasets with respect to in situ measurements, including ET, P, and SM data. (**B**) Performances of BERTH and the other published datasets with different land cover types, normalized by the fifth-generation European Centre for Medium-Range Weather Forecasts reanalysis (ERA5-Land). [Normalized root mean square deviation (RMSD) and correlation coefficient (CC) are calculated as the ratio of a dataset’s metric to that of ERA5-Land, with both evaluated against in situ measurements.] (**C**) Improvements of BERTH and other published datasets with respect to in situ measurements with complex terrain conditions (for each station where slope > 5°). The baselines of the P and SM evaluations are IMERG Final Run and SMAP (9 km), respectively. d, day.

The BERTH model capitalizes on all available model inputs, including multichannel remote sensing reflectance, meteorological conditions, and topographical variables that are related to water cycle. ET, P, and SM are estimated simultaneously, and an evaluation conducted on independent datasets reveals that the BERTH model is robust and superior to the existing approaches (tables S1 to S3). As shown in Fig. 2A, the BERTH model achieves higher correlation coefficient (CC) and lower root mean square deviation (RMSD) metrics computed relative to in situ measurements than the other widely used remote sensing–based quantification models, which are considered the best. The integration of multisource data is pivotal for enhancing the accuracy of retrieved water cycle variables. For instance, the Integrated Multi-satellitE Retrievals for Global precipitation measurement (IMERG) Final product, refined through calibration with monthly ground-based P, exhibits superior performance over the IMERG Early version. Similarly, by incorporating P and SM data, ETMonitor captures diurnal ET variations more robustly than the MOD16A2 product. By leveraging both remote sensing and in situ observations, the BERTH model demonstrates substantial advancements in monitoring continuous land surface hydrological processes. Such improvements underscore the necessity of data fusion in mitigating the sensitivities inherent in single-source satellite observations (fig. S8).

We further analyze the performances of all those models with various land cover types (Fig. 2B). The land component of the fifth-generation European Centre for Medium-Range Weather Forecasts reanalysis (ERA5-Land) that provides all water cycle components is selected as the baseline for calculating the normalized RMSD (RMSD_model_/RMSD_ERA5-Land_) and normalized CC (CC_model_/CC_ERA5-Land_) (*41*). Figure 2B shows the relative performances achieved by several water cycle datasets with different land cover types, including forests, shrublands, savannas, grasslands, and croplands. The BERTH model substantially improves the CC by ~14% and reduces the RMSD by ~20%, whereas the accuracy differences among the other datasets are relatively small. Unlike the BERTH model that performs well with almost all land cover types, ERA5-Land and the other remote sensing methods are satisfactory only in some cases. (i) The SM retrieved from microwave remote sensing data is more accurate in areas with smaller canopy thicknesses, such as grasslands, than the simulated SM of ERA5-Land is, but the model does not perform as well in forests. (ii) In areas with heavy human activity such as croplands, the remotely sensed SM data are more reliable than ERA5-Land is, so the latter cannot be used to quantify human irrigation schedules (*42*). (iii) Regarding ET and P, which are closely related to the atmosphere, ERA5-Land performs better than satellite-based remote sensing datasets because ERA5-Land assimilates ground-based temperature, wind speed, and radiation data for retrieving ET and P. Overall, the BERTH model achieves the best performance for all components with almost all of the land cover types, regardless of whether they are forests or croplands (table S4).

Mountainous areas have large elevation variations, affecting the movement of air masses and determining the distributions of P and SM (*43*). We evaluate the performance of the BERTH model in terms of retrieving P and SM values in mountainous areas using an independent in situ dataset. Figure 2C illustrates the performance gains of BERTH across different water cycle variables: For P, improvements are shown for BERTH and IMERG Early Run relative to IMERG Final Run (defined as RMSD_model_ − RMSD_IMERG Final_ and CC_model_ − CC_IMERG Final_); for SM, improvements are shown for BERTH and Advanced Microwave Scanning Radiometer for Earth Observing System with Multi-Channel Collaborative Algorithm (AMSR-E/2, MCCA) against Enhanced Soil Moisture Active Passive (SMAP) (9 km) (defined as RMSD_model_ − RMSD_SMAP_ and CC_model_ − CC_SMAP_) for each site with slope > 5°. The BERTH model has better performance than the famous IMRGE Final and Enhanced SMAP (9 km) models, with a higher CC and a lower RMSD at the same time. For P, BERTH outperforms the popular IMERG Final Run model, although it is not further calibrated with monthly gauge-based P data. Regarding SM, the BERTH model outperforms Enhanced SMAP (9 km), although its pretraining dataset acquired from AMSR-E/2 (MCCA) is not as good as that of Enhanced SMAP (9 km). Moreover, satellite-based P and SM datasets are derived from microwave sensors with coarse spatial resolutions (*2*), which are not suitable for supporting water-related research or management in mountainous areas. In contrast, the BERTH model driven by big Earth data has the advantages of both strong accuracy and high resolutions, which is highly valuable for conducting research in areas with complex terrain conditions.

### The BERTH model demonstrates robust performance at high spatiotemporal scale

**Fig. 3.**
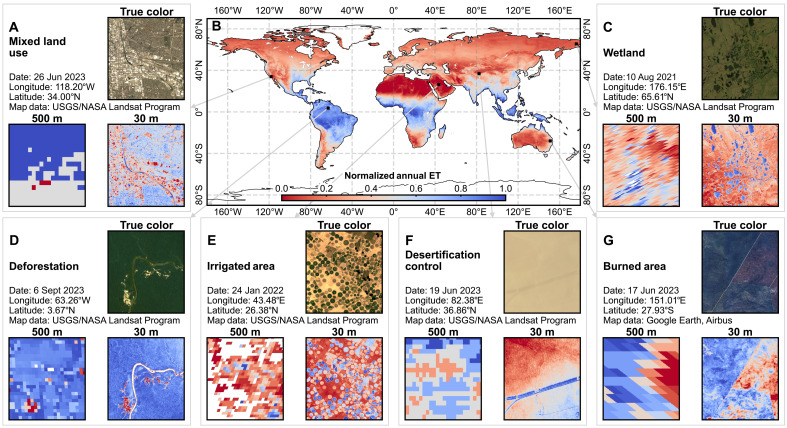
Resolving fine-scale water cycle heterogeneity across diverse global landscapes. The central map (B) displays the global pattern of ET in 2020. Surrounding panels [(A) and (C) to (F)] compare the spatial details of ET retrieval between the 500-m MODIS-based product and the 30-m BERTH product across six representative landscape archetypes. Each panel includes a true-color reference image (top), the coarse-resolution MODIS baseline (bottom left), and the high-resolution BERTH retrieval (bottom right). (**A**) Mixed impervious and vegetated surfaces in an urban interface (Los Angeles, USA); (**B**) spatial patterns of global ET in 2020; (**C**) fragmented wetland mosaics (Siberia, Russia); (**D**) anthropogenic deforestation boundaries from mining (Yanomami, Amazon); (**E**) circular patterns of center-pivot irrigation (Qassim, Saudi Arabia); (**F**) linear ecological restoration belts for desertification control (Xinjiang, China); and (**G**) sharp transition zones in prescribed burn areas (Queensland, Australia). The image entitled “True color” in (G) is obtained from Google Earth historical imagery (credit: Airbus), and the other images entitled “True color” in (A) and (C) to (F) are derived from Landsat imagery [US Geological Survey (USGS)/NASA Landsat Program].

The spatial heterogeneity of terrestrial water cycle, especially ET, is frequently obscured by the coarse resolution of traditional satellite–based products, particularly in regions undergoing rapid anthropogenic modification or characterized by fragmented ecosystems. Figure 3 illustrates this critical scale mismatch by contrasting the 500-m estimates based on the Moderate Resolution Imaging Spectroradiometer (MODIS) with our 30-m BERTH product across six globally distinct landscape archetypes. While the global map captures the broad latitudinal gradients of ET, the case study insets reveal that coarse-resolution pixels systematically fail to resolve the fine-scale hydrological processes driven by local land-use dynamics.

This loss of fidelity is most pronounced in landscapes dominated by geometric anthropogenic features. In highly heterogeneous urban matrices like Los Angeles (Fig. 3A), the 30-m resolution successfully characterizes the complex spatial details of impervious surfaces and urban vegetation, which remains unresolved in coarser datasets. The intricate mosaic of water bodies and vegetation in the Siberian wetlands (Fig. 3C) is preserved only at high resolution. Sharp transition boundaries resulting from human intervention, such as the sinuous deforestation scars caused by gold mining in the Amazon (Fig. 3D) and the distinct linear firebreaks of prescribed burns in Australia (Fig. 3G), are blurred or entirely lost at the 500-m scale. In the arid oasis agriculture of Saudi Arabia (Fig. 3E), the precise circular boundaries of center-pivot irrigation systems, delineated in the BERTH product, are homogenized into indistinct clusters by the spatial averaging of MODIS. In the Taklamakan Desert (Fig. 3F), the narrow vegetation belt planted for windbreak and sand fixation along the railway is visible as a continuous high-ET corridor in the BERTH product but is virtually undetectable in MODIS due to the mixed-pixel effect. These comparisons demonstrate that the aggregation bias inherent in 500-m data obscures pronounced local variability, suggesting that high-resolution monitoring is a prerequisite for accurately quantifying water fluxes in spatially complex environments.

**Fig. 4.**
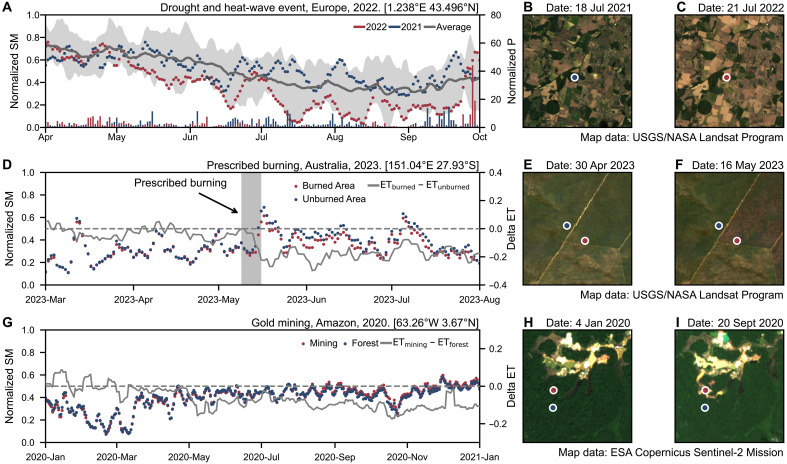
Temporal patterns of hydrological responses to climatic extremes and human activities. Drought events [(A) to (C)], fires [(D) to (F)], and mining [(G) to (I)]. (**A**) Time series of normalized SM and P during the 2022 European megadrought (purple), the 2021 baseline (blue), and their multiyear daily average (gray). True-color snapshots compare the landscape in July 2021 (**B**) and the desiccated state in July 2022 (**C**). (**D**) Hydrological impact of prescribed burning in Queensland, Australia, 2023. The gray shaded area marks the burn event, followed by an immediate divergence in SM between the burned area (purple) and unburned area (blue), and a corresponding drop in the ET. True-color snapshots compare the landscape before (**E**) and after (**F**) the fire. (**G**) Impact of gold mining on rainforests in Yanomami, Amazon. The time series tracks the ET and SM dynamics in mining areas (purple) relative to adjacent intact forest (blue). True-color snapshots show the expansion of mining pits and loss of forest cover in January 2020 (**H**) and September 2020 (**I**), respectively. All ET (lines), P (bars), and SM (dots) values are derived from BERTH 30-m retrievals, demonstrating the model’s ability to parse complex temporal patterns. The images in (B), (C), (E), and (F) are derived from Landsat imagery [US Geological Survey (USGS)/NASA Landsat Program]. The images in (H) and (I) are derived from Sentinel-2 imagery [European Space Agency (ESA)/Copernicus Sentinel-2 Mission].

Beyond its superior spatial fidelity, the BERTH framework also demonstrates exceptional sensitivity to the temporal dynamics due to both climate and human activities. As illustrated in Fig. 4 (A to C), the model effectively captures the 2022 European extreme drought event. The 30-m SM trajectories in 2022 reveal a severe and sustained departure from the multiyear climatological mean, especially during the summer without P. This situation continued until the heavy rainfall event in October brought it to an end. In the prescribed burn regions of Australia (Fig. 4, D to F), BERTH captures the sudden changes before and after the implementation of fires. ET in the burned area was markedly lower than that in the adjacent unburned area. Similarly, the BERTH model tracks the variation of SM and ET associated with deforestation due to gold mining in the Amazonian regions (Fig. 4, G to I). By accurately capturing these temporal patterns, ranging from interannual signals of large-scale heat waves to the sudden changes due to fire and deforestation, BERTH provides a robust tool for monitoring the global water cycle and its feedback mechanisms, especially in an era of accelerating environmental change.

## DISCUSSION

The transition from coarse- to high-resolution water cycle retrieval represents a fundamental leap in modeling and analyzing the terrestrial water cycle. BERTH offers critical insights for refining Earth system models that have historically struggled with the parameterization of subgrid heterogeneity. This capability is particularly urgent in the context of a warming climate, where the intensification of the water cycle is driving a higher frequency of extreme events (figs. S1 and S2); capturing the onset of localized anomalies, such as agricultural flash droughts or rapid wetland degradation, requires resolving the spatial variability that coarse sensors inevitably obscure. However, limitations remain, primarily regarding the trade-off between spatial detail and temporal continuity, as well as the susceptibility of optical methods to cloud contamination. Future efforts must therefore focus on the synergistic integration of multisource observations, fusing high-resolution optical data with all-weather microwave sensing, and the application of deep learning paradigms to reconstruct spatiotemporal dynamics of the entire Earth systems, ultimately bridging the gap between local ecohydrological processes and global climate projections. Moving forward, a critical research frontier lies in the development of physics-informed data-driven frameworks that explicitly incorporate fundamental constraints, such as the terrestrial water balance. For variables such as groundwater and RO, where ground-based field observations are lacking, incorporating physical mechanism constraints is the only feasible approach. By integrating observations across a broader spectral range and embedding rigorous physical laws into the deep learning architecture, future iterations of BERTH can achieve a more holistic reconstruction of the global water cycle, thereby enhancing both the physical consistency and the structural integrity of the retrieved water cycle variables.

Cloud contamination and satellite orbits limit the temporal continuity of high-resolution remote sensing techniques in the optical and thermal bands. Many postprocessing-based spatiotemporal interpolation algorithms, such as linear interpolation, Kalman filtering, and deep neural networks (*44*, *45*), were developed to obtain continuous data in time and space. However, in areas with high spatial heterogeneity (e.g., those with human activities and extreme climate events), interpolation methods introduce extra uncertainties. Researchers studying earth sciences often must address discontinuous remote sensing data. Transformers, which use self-attention mechanisms to capture the relationships among words, have achieved great success in NLP tasks (*46*). BERT-like models use a masked language modeling approach to effectively highlight relevant contextual information by adding mask labels (*29*). Similarly, many masks are contained in the input remote sensing time series and natural language sentences at the same time. By recognizing such great parallels between the hydrological information encoded in remote sensing data and the semantic information encoded in natural language, BERTH is developed to quantify land surface hydrological information. The self-attention mechanism of the model adeptly captures temporal variations, thoroughly uncovering the encoded hydrological dynamics although satellites do not overpass (tables S5 and S6) or some input variables exhibit notable retrieval errors (fig. S8). By discretizing long-term remote sensing observations at daily scales, analogous to words in a sentence, we use the end-to-end transformer encoder to analyze and extract embedded water cycle processes from the remote sensing data.

## MATERIALS AND METHODS

### BERTH architecture

Building upon the architectural framework described in the “Transformer-based architectures driven by remote sensing enable water cycle modeling” section, this section details the computational implementation and technical specifications of BERTH. An overview of the model architecture of BERTH is presented in Fig. 1C. It first splits the input time series with a fixed length of 500 days into individual tokens at the daily scale. Each token has six remote sensing features [blue, green, red, near-infrared (NIR), and two shortwave infrared (SWIR) bands] and eight atmospheric features [10-m eastward/northward (U/V) components of wind speed, 2-m air temperature, 2-m temperature at the dew point, surface pressure, and surface downward solar/thermal radiation]. These tokens and their terrain features (elevations, slopes, and aspects) are subsequently projected to the same 256-dimensional hidden space as that of the transformer encoder via a linear transformation. A learnable relative date embedding is added to the projected features to retain their temporal information. A transformer encoder with MSA is used to capture the hidden information of sparse remote sensing time series, with another standard transformer encoder used for dense atmospheric forcing data. Furthermore, the hidden remote sensing, atmospheric forcing, and terrestrial features are concatenated and then processed by a third transformer encoder to capture the potential relationship between the remote sensing and hydrological information. Each transformer encoder of the BERTH model has three encoder layers that consist of a four-head self-attention module and a multilayer perceptron (MLP) with the hidden dimension of 2048 (*46*). Both the attention and MLP modules incorporate residual connections and layer normalization, consistent with the standard transformer architecture. Last, at the output module, the hidden features, including ET, P, SM, and RO, are projected to the hydrological information dimension by a linear transformation matrix with the dimension of 768 × 4. In this study, the BERTH model is implemented with the standard modules in the open-source PyTorch library and trained on a cluster with four NVIDIA A100 GPUs.

### Inputs of the BERTH model

The inputs of the BERTH model consist of three parts: remote sensing, atmospheric forcing, and terrain features. (i) Surface reflectance data acquired from Terra MODIS and Aqua MODIS are combined via the simple averaging method, whereas surface reflectance data obtained from Landsat-7 ETM+, Landsat-8 OLI, Landsat-9 OLI2, and Sentinel-2 MSI are harmonized after performing spectral bandpass adjustments, as done by Claverie *et al.* (*47*). These sensors share similar bands in the atmospheric window, namely, red, green, blue, NIR, and two SWIR bands, which are selected as the remote sensing features of the BERTH inputs (*39*, *40*). (ii) ERA5-Land is downscaled with bilinear interpolation to the same spatial resolution as that of the remotely sensed surface reflectance data. ERA5-Land downward solar radiation, downward thermal radiation, surface pressure, the 10-m U component and V component of wind, the 2-m air temperature, and the 2 m dew-point temperature are selected as the atmospheric forcing features of the BERTH inputs (*40*). (iii) The MERIT DEM is upscaled to the same spatial resolution as that of MOD09GA/MYD09GA. The elevation and further calculated aspect and slope derived from the MERIT DEM and Copernicus DEM GLO-30 are selected as the terrain features of the BERTH inputs. All of these data are preprocessed via the Google Earth Engine.

The integration of multisource data in BERTH leverages the distinct yet complementary roles of remote sensing, meteorological, and topographic inputs. (i) Remote sensing observations provide essential constraints on the spatiotemporal heterogeneity of the soil-plant system. Spectral reflectance is directly coupled with water cycle variables; for instance, SM modulates the absorption coefficients of electromagnetic waves, while root-zone water availability governs vegetation density and phenological traits. (ii) Meteorological forcing captures large-scale atmospheric states—including solar radiation, temperature, humidity, and wind speed—which act as the primary drivers of surface ET. By assimilating these near-surface atmospheric dynamics, BERTH effectively reconstructs the terrestrial water cycle, enabling the simultaneous retrieval of ET, P, and SM. (iii) Topographic features dictate the redistribution of water and energy across complex terrains. Specifically, slope is a critical determinant of surface RO routing, while aspect serves as a primary modulator of net radiation budgets within microenvironments. Furthermore, incorporating topography accounts for terrain-induced effects on satellite observations, thereby enhancing the precision of quantitative geophysical inversions.

### Pretraining and fine-tuning paradigms

It is not sufficient to directly train a large model on the available in situ data. We train the BERTH model that contains ~20 million trainable parameters through two supervised steps: Pretraining with the spatial resolution of 500 m on conventional published datasets and fine-tuning with the spatial resolution of both 500 and 30 m on in situ data (fig. S5), using 620 million tokens and 37 million tokens, respectively.

In the pretraining step, we randomly select 10,000 points per land cover type contained in the global mosaic of the International Geosphere-Biosphere Programme classification from MCD12Q1 for training, as shown in fig. S7. For each selected point, ET, P, SM, and RO data are obtained from conventional global datasets: IMERG Final Run (version 7B), AMSR-E/2 (MCCA), ETMonitor, and Global Reach-level Flood Reanalysis (GRFR), respectively. IMERG Final Run is the latest P product published by the Global Precipitation Measurement (GPM) mission. ETMonitor estimates global daily ET values with a spatial resolution of 1 km. AMSR-E/2 (MCCA) was developed through a multichannel collaborative algorithm with rigorous physical models. The GRFR dataset provides global RO estimates with a spatial resolution of 0.05°. These water cycle datasets share a temporal resolution of 1 day and have the best-in-class spatial resolutions. Their spatial resolutions are unified to 500 m on the basis of the nearest neighbor sampling method. The weights of the BERTH model are optimized by a standard adaptive moment estimation (Adam) optimizer, which minimizes the overall mean squared error loss induced on all these water cycle data (*48*). The learning rate is initialized as 1 × 10^−5^ and then dynamically modulated via a decay scheduler that monitors the average training loss.

In the following fine-tuning step, a subset of publicly available in situ data observed by gauges, eddy covariance systems and SM sensors are used for model refinement purposes. SC-EARTH uses raw station data acquired from the Global Historical Climatology Network–Daily and Global Surface Summary of the Day datasets and provides a unified station repository after performing station merging and strict quality control operations. The International Soil Moisture Network is an international cooperative that aims to establish and maintain a global in situ SM database (ISMN). FLUXNET is an international community that uses an eddy covariance system to measure the fluxes of the Earth’s surface, including those of carbon, water, and energy. FLUXNET2015 and FLUXNET-CH4 are the two main products of FLUXNET. AmeriFlux and EuroFlux are also combined to provide updated in situ LE data with the same format as that of FLUXNET2015. First, we randomly separate all in situ stations into three groups according to their land cover types: training (70%), validation (20%), and testing (10%) groups. We subsequently freeze almost all of the model weights, including those of the embedding and transformer encoder modules. The last linear generator is trained individually for each water cycle variable. Last, the whole model is fine-tuned with a smaller learning rate. An early stopping strategy based on an independent validation set was used to prevent overfitting. Given that in situ measurements for RO are inaccessible, BERTH was trained using GRFR as the RO target in this step. To further ensure physical consistency, the retrieved RO was subjected to a water balance closure correction at the annual scale, constrained by the corresponding P and ET estimates. This two-step training approach ensures that the model is robustly trained using sufficient data.

Some well-known datasets are selected for conducting comparison assessments. IMERG Early Run is a satellite-based P dataset derived from GPM data using radiative transfer models. MOD16A2 (version 006) is the global terrestrial ET product estimated using MODIS data and the Penman-Monteith equation. MOD16A2 provides total ET and potential ET data with a spatial resolution of 500 m and a temporal resolution of 8 days. The SMAP mission has been measuring SM since 2015 and provides global SM products with advanced accuracy. Its enhanced versions, with spatial resolutions of 9 and 1 km, are used in this study.
