# Supplementary material for: Transforming global water cycle observations via synergistic AI and remote sensing
Source: Sci Adv. 2026 Jun 17;12(25):eaef3610. doi: 10.1126/sciadv.aef3610 (PMC13274585; doi:10.1126/sciadv.aef3610)
Supplement: Supplementary file 1 — Supplementary Text S1 to S5 Figs. S1 to S8 Tables S1 to S7 [file sciadv.aef3610_sm.pdf]

Science Advances
AAAS

Supplementary Materials for

Transforming global water cycle observations via synergistic AI and remote sensing

Zhaoyuan Yao *et al.*

Corresponding author: Yaokui Cui, yaokuicui@pku.edu.cn

Sci. Adv. **12**, eaef3610 (2026)
DOI: 10.1126/sciadv.aef3610

This PDF file includes:

Supplementary Text S1 to S5
Figs. S1 to S8
Tables S1 to S7

1. Evaluating model performances based on ground-truth-data

‘Training’ is used when updating model weights, while ‘Validation’ is used when adapting hyperparameters. ‘Evaluation’ is completely independent of the above two sets. Detailed performances of P, SM and ET can be found in Table S1, Table S2, and Table S3, respectively. Table S4 shows BERTH’s performance over different land cover types. Evaluations when satellites overpass in situ stations or not with the spatial resolution of 500 m and 30 m are show in Table S5 and Table S6, respectively.

2. Seasonal and interannual variability

2.1 El Niño–Southern Oscillation

El Niño–Southern Oscillation (ENSO) is one of the most famous global climate phenomena. When El Niño occurs, sea surface temperatures in the eastern Pacific Ocean anomalously increase. A widespread and prolonged El Niño event occurred in 2015. Fig. S1 illustrates the difference in annual precipitation for 2015 relative to the average annual precipitation of the 2010s, derived from BERTH and IMERG, respectively. El Niño events lead to increased precipitation in regions such as southeastern China and the southern United States, while causing decreased precipitation in areas like Southeast Asia and the Amazon. This result indicates that the BERTH model is capable of capturing interannual variations in precipitation.

2.2 The Great Wildebeest Migration in Eastern Africa

In Eastern Africa, the Great Wildebeest Migration occurs in dry seasons (from May to October). The drying of the Serengeti (Tanzania) grasslands and associated decrease in evapotranspiration drive animal migration to the Masai Mara (Kenya), where vegetation is lush and evapotranspiration is higher. When the rainy season returns, animals migrate back to the vast Tanzanian grasslands. Fig. S2 illustrates the differences in evapotranspiration between dry-season (July) and wet-season (March) grasslands. This result indicates that the BERTH model is capable of capturing seasonal variations in evapotranspiration.

2.3 Runoff-to precipitation patterns

Runoff generation is primarily driven by short-duration intense precipitation. The spatial distributions of runoff and precipitation exhibit a high degree of correlation at the daily scale. Analyzing the spatial synchronicity between precipitation and runoff serves to validate the physical consistency of the model's inversion results. Here, we examined the physical coupling between precipitation and runoff to ensure the internal consistency of the model (see Fig. S3). A critical observation is that BERTH's runoff generation accurately tracks the seasonal migration of global rain-belts. The model captures the synchronized movement of precipitation peaks and runoff responses, particularly in monsoon-driven regions and the Intertropical Convergence Zone.

3. Comparison with other dataset

Physical models contain empirical equations, approximations and simplifications. Thus, their performances are affected by the spatial scale of the input data. Here, we visually compare the spatial patterns of the BERTH outputs with those of the other datasets at both global and field scales.

3.1 Global scale

At the global scale, precipitation is the main driver of the famous hydrological models, such as the variable infiltration capacity (VIC) model and the Global Land Data Assimilation System (GLDAS). We demonstrate the ability of the BERTH model to reflect global annual patterns of P, surface SM, ET and runoff relative to those of Integrated Multi-satellitE Retrievals for Global Precipitation Measurement (IMERG) Final Run, Enhanced Soil Moisture Active Passive (SMAP) (9 km), MOD16A2 and Global Reach-level Flood Reanalysis (GRFR). As shown in Fig. S4, BERTH has similar global patterns to those of the other products. Sufficient precipitation and high evaporation levels are contained in areas with tropical rainforests, such as Southeast Asia and the Amazon. The prevailing westerly winds strongly transport water vapor from the ocean to land in land–sea junction areas, such as the west coast of Canada. The water vapor generated by the monsoons of South Asia results in considerable

precipitation and runoff on the southern side of the Tibetan Plateau. Although it operates in a pixel-by-pixel manner, the BERTH model accurately describes the global water distribution and the supply and consumption levels in different regions.

We further compare runoff derived from BERTH and other reanalysis models. As illustrated in Fig. S5, the spatial distribution of BERTH runoff exhibits high consistency with both ERA5-Land and GLDAS 2.1 across all major climate zones. BERTH accurately captures the macro-scale hydrological patterns, including the high-runoff signatures in tropical regions (e.g., the Amazon and Southeast Asia), the near-zero runoff in arid and semi-arid zones (e.g., the Sahara and Central Asia), and areas with large annual runoff anomalies (e.g., southern Brazil's exceptionally low runoff in 2020). The synchronization of these spatial gradients and seasonal fluctuations—despite the fundamental differences between our AI-driven approach and the physics-based land surface models—confirms the robustness and cross-product reliability of the BERTH framework.

3.2 Field scale

The BERTH model pretrained at the global scale can be easily fine-tuned for applications conducted at the field scale. At this scale, human activities such as irrigation regulate hydrological processes in croplands and lead to strong spatial heterogeneity. Actual ET over irrigated cropland is one of the major sources of water consumption and determines how much irrigation water should be applied to fields. Accurately estimating ET in irrigated croplands is a considerable challenge. We fine-tune BERTH at a spatial resolution of 30 m to address the challenge of quantifying the water budget at the field scale. Several typical high-resolution remote sensing sensors, including ETM+ onboard Landsat-7, OLI onboard Landsat-8, OLI2 onboard Landsat-9, and SLI onboard Sentinel-2, are combined to obtain more frequent revisits. OpenET is a continental-scale ET mission that makes comprehensive use of multisource data and integrates six models to estimate ET at a high spatial resolution (30 m). Fig. S6 shows the relative ET in September 2022 in a randomly selected crop field in the middle of the U.S., where large centre-pivot

irrigation equipment is used. At the field scale, the variation exhibited by atmospheric data is relatively small over flat croplands. Therefore, the differences between the ET of irrigated croplands and non-irrigated areas (shown in Fig. S4) are captured mainly by remote sensing data. The BERTH model directly uses high-resolution optical remote sensing data and draws clear boundaries, whereas OpenET takes interpolated thermal infrared data as its main inputs, and its results are blurred at the boundaries of the croplands. Similarly, the BERTH model is also efficient at quantifying P, SM and RO at the field scale, which is still a considerable challenge for the current physical models. By coupling remote sensing time series data, the BERTH model can accurately estimate the water cycle at high spatial resolutions and provide more spatial details than the other advanced models can provide.

4. Dataset used for this study

Dataset used in this study and their roles are listed in Table S7. Locations of points sampled in pre-training step and collected in fine-tuning step are shown in Fig. S7.

5. Sensitivity analysis

The integration of multi-source datasets necessitates a rigorous evaluation of how input uncertainties propagate through the BERTH framework. Our sensitivity analysis reveals that the model responses vary across different hydrological variables. Specifically, air temperature serves as a primary driver for the overall water cycle intensity, while P estimates exhibit heightened sensitivity to downward longwave radiation and surface pressure. ET fluxes, conversely, are more responsive to variations in dew point temperature, reflecting the model's capacity to capture the moisture demand of the atmosphere.

To assess the operational robustness of BERTH, we subjected the input forcing to extreme noise scenarios. For instance, introducing a 10% maximum random noise to air temperature—equivalent to an absolute error of ~14 K, which far exceeds the standard 1-2 K uncertainty range of ERA5-Land—did not lead to catastrophic failures in output quality. This stability suggests that the synergistic architecture of BERTH effectively avoids individual input errors. By learning the inherent physical correlations between multiple variables through its self-attention

mechanism, the model can cross-reference information from stable channels to compensate for degraded inputs. This multi-variable buffering mechanism is essential for global applications where the quality of forcing data may be inconsistent across space and time.

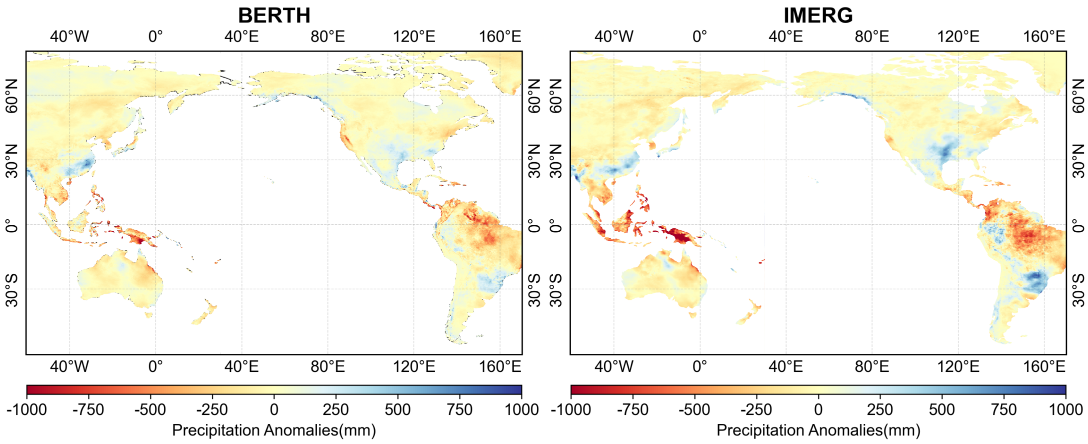

Fig. S1 Difference between annual precipitation in 2015 and average annual precipitation during 2010-2019, derived from BERTH (left) and IMERG (right).

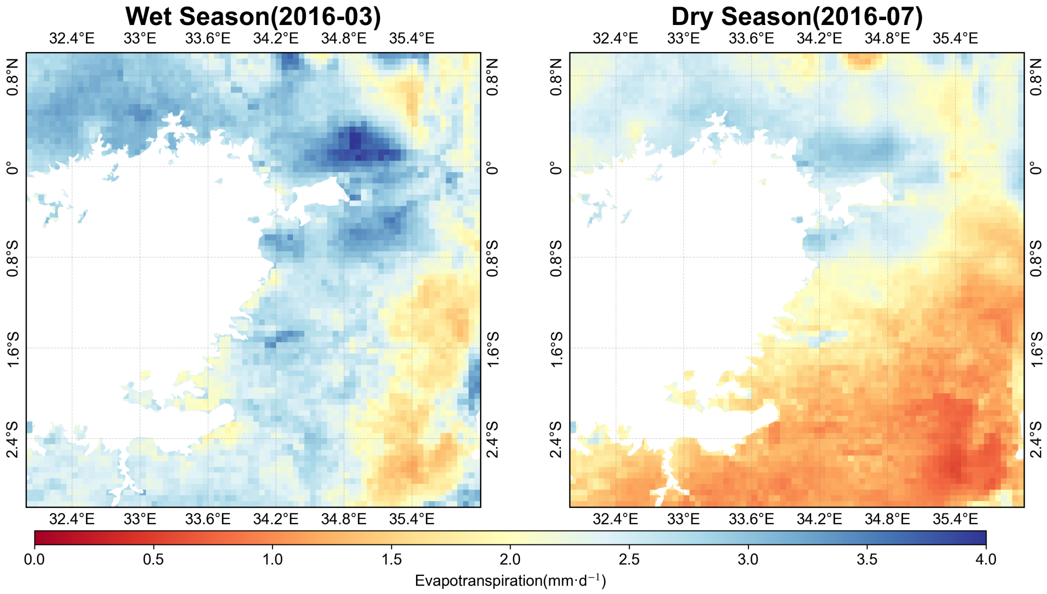

Fig. S2 Daily evapotranspiration of grasslands in East Africa, derived from BERTH.

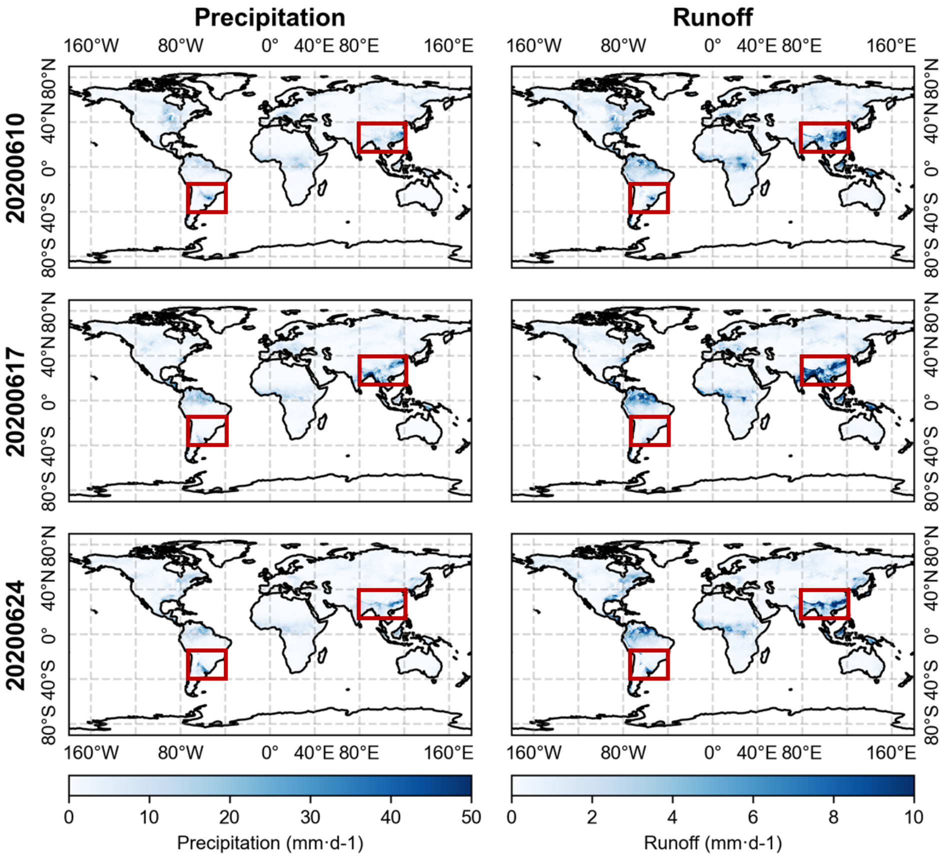

Fig. S3 Spatiotemporal coupling between precipitation and runoff generated by BERTH.

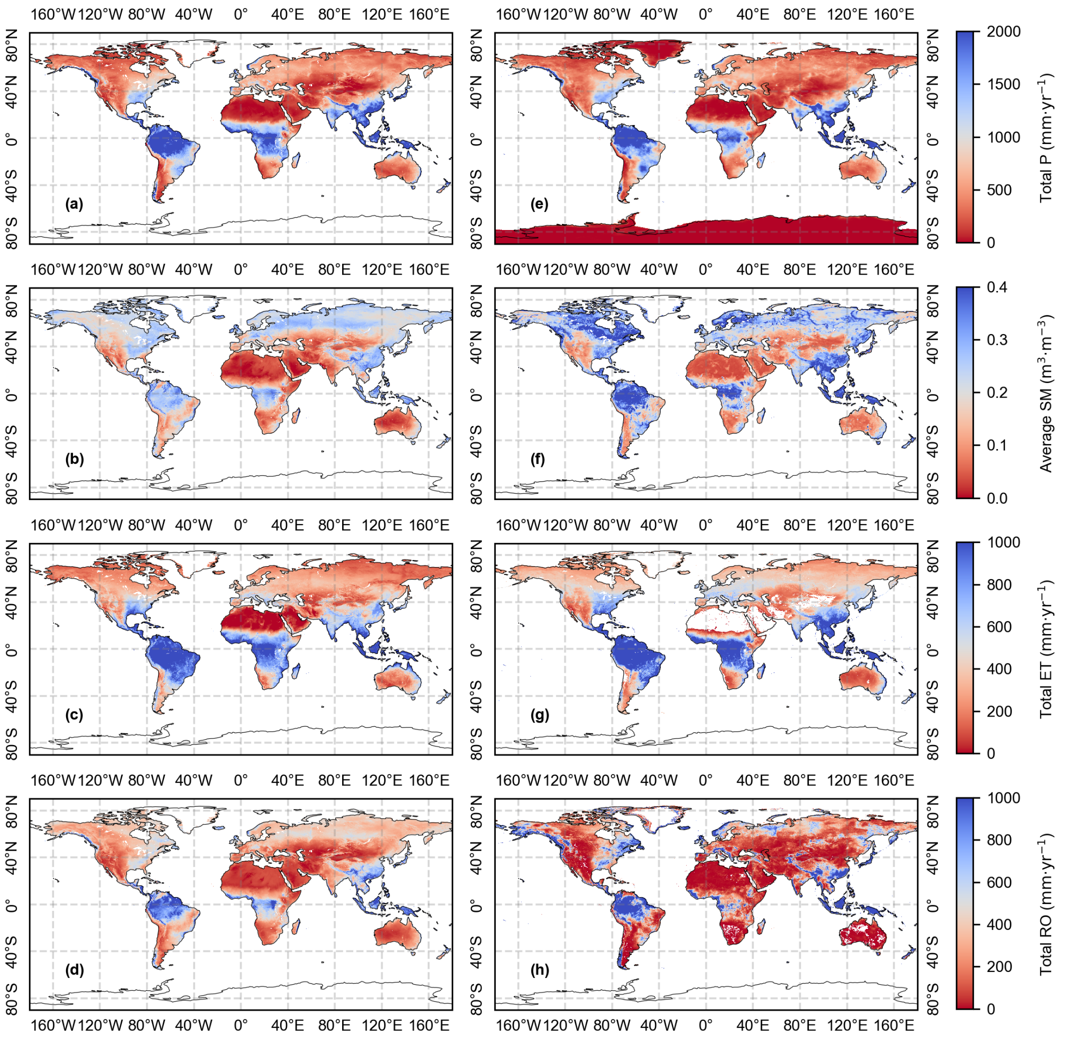

Fig. S4 Global annual hydrology in 2022 estimated by BERTH (a-d), IMERG Final Run (e), SMAP (f), and GRFR (g).

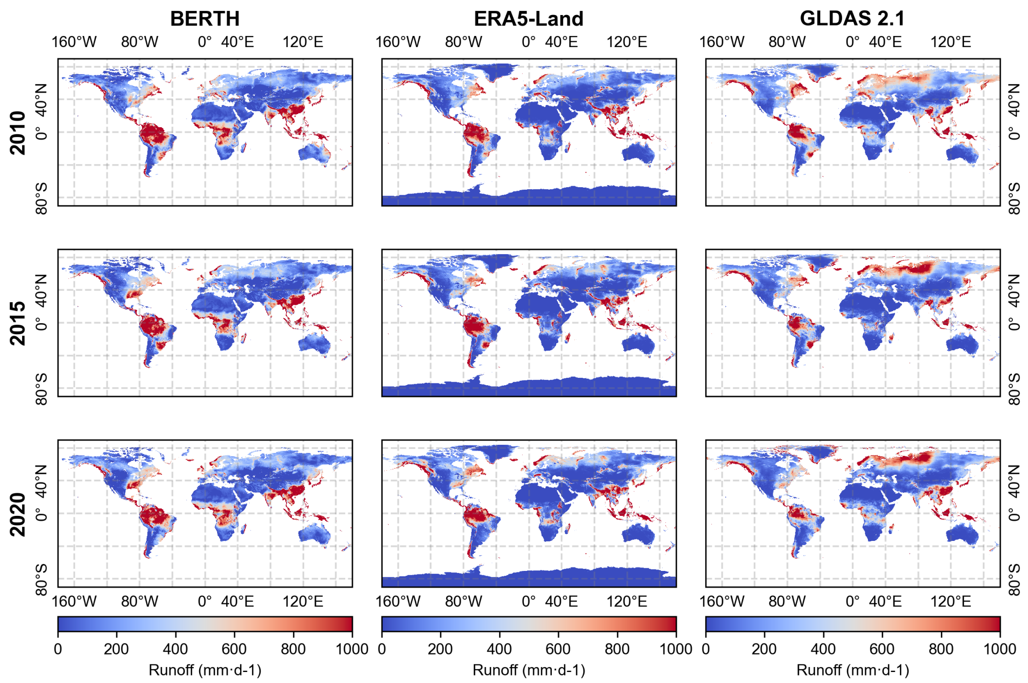

Fig. S5 Global annual hydrology in 2022 estimated by BERTH (a-d), IMERG Final Run (e), SMAP (f), and GRFR (g).

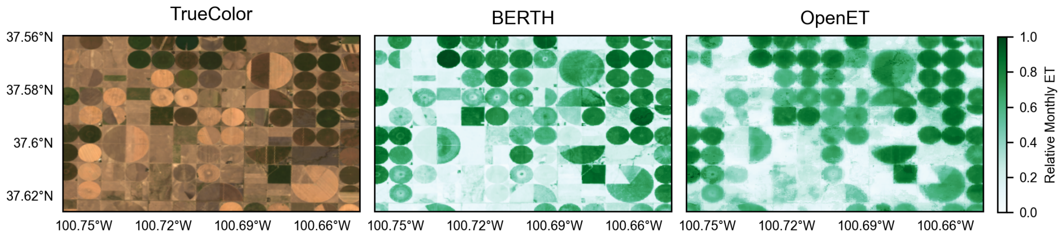

Fig. S6Actual ET at the field scale in the middle of the U.S. in September 2022.

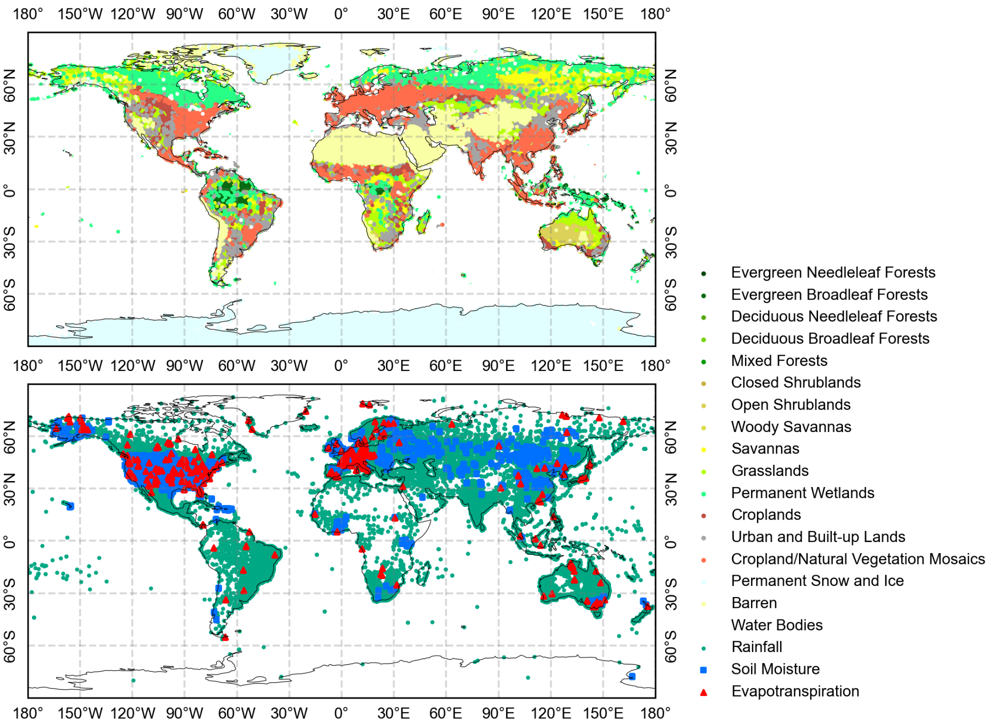

Fig. S7 Locations of (top) the points used for pretraining and (bottom) the global in situ stations employed for fine-tuning and evaluation purposes.

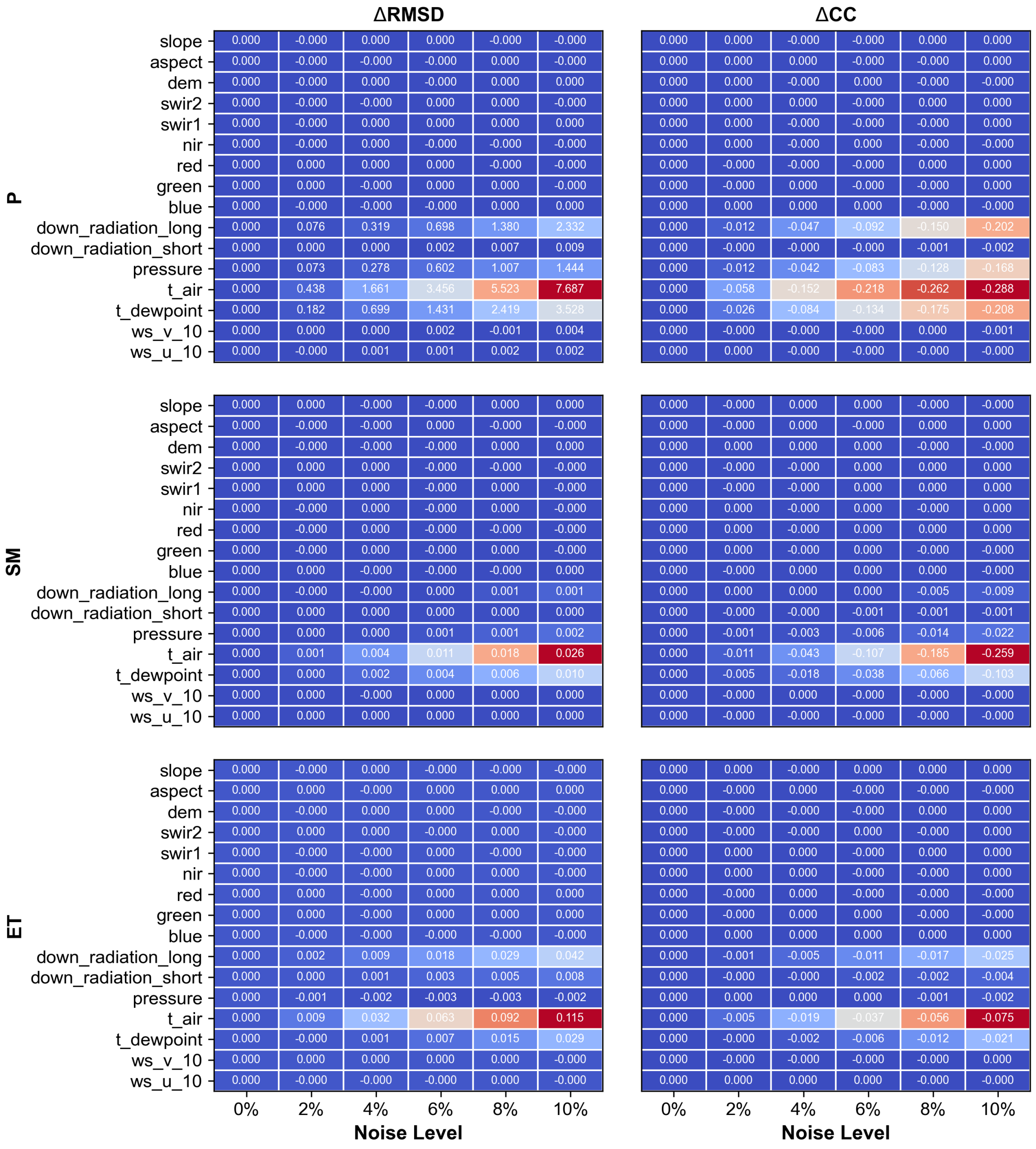

Fig. S8 Sensitivity analysis of BERTH

Table S1. Performances of different P datasets.

Dataset	Group	Overall RMSD	Overall CC	Median RMSD	Median CC
IMERG Final Run (V7B)	Training	7.81 mm·d^{-1}	0.46	6.55 mm·d^{-1}	0.45
	Validation	7.72 mm·d^{-1}	0.47	6.52 mm·d^{-1}	0.48
	Evaluation	7.77 mm·d^{-1}	0.47	6.46 mm·d^{-1}	0.48
IMERG Early Run (V6)	Training	9.49 mm·d^{-1}	0.39	8.04 mm·d^{-1}	0.36
	Validation	9.46 mm·d^{-1}	0.40	8.23 mm·d^{-1}	0.40
	Evaluation	9.52 mm·d^{-1}	0.40	8.20 mm·d^{-1}	0.39
BERTH-30m	Training	6.73 mm·d^{-1}	0.55	**5.32** mm·d^{-1}	**0.52**
	Validation	6.81 mm·d^{-1}	0.52	5.40 mm·d^{-1}	0.51
	Evaluation	6.83 mm·d^{-1}	0.52	5.36 mm·d^{-1}	0.50
BERTH-500m	Training	**6.54** mm·d^{-1}	**0.56**	5.35 mm·d^{-1}	0.51
	Validation	**6.57** mm·d^{-1}	**0.54**	**5.38** mm·d^{-1}	**0.52**
	Evaluation	**6.57** mm·d^{-1}	**0.54**	**5.34** mm·d^{-1}	**0.52**

Table S2. Performances of different SM datasets.

Dataset	Group	Overall RMSD	Overall CC	Median RMSD	Median CC
AMSR-E/2	Training	0.113 m^3·m^{-3}	0.43	0.104 m^3·m^{-3}	0.43
	Validation	0.112 m^3·m^{-3}	0.48	0.100 m^3·m^{-3}	0.46
	Evaluation	0.110 m^3·m^{-3}	0.48	0.096 m^3·m^{-3}	0.49
SMAP_9km	Training	0.111 m^3·m^{-3}	0.49	0.087 m^3·m^{-3}	**0.65**
	Validation	0.109 m^3·m^{-3}	0.52	0.087 m^3·m^{-3}	**0.63**
	Evaluation	0.119 m^3·m^{-3}	0.49	0.083 m^3·m^{-3}	**0.65**
SMAP_1km	Training	0.112 m^3·m^{-3}	0.50	0.086 m^3·m^{-3}	0.59
	Validation	0.111 m^3·m^{-3}	0.52	0.088 m^3·m^{-3}	0.59
	Evaluation	0.119 m^3·m^{-3}	0.50	0.086 m^3·m^{-3}	0.60
BERTH-30m	Training	**0.080** m^3·m^{-3}	0.71	**0.073** m^3·m^{-3}	0.60
	Validation	0.093 m^3·m^{-3}	0.62	**0.081** m^3·m^{-3}	0.58
	Evaluation	**0.093** m^3·m^{-3}	**0.63**	**0.077** m^3·m^{-3}	0.59
BERTH-500m	Training	0.081 m^3·m^{-3}	**0.72**	0.078 m^3·m^{-3}	0.55
	Validation	**0.090** m^3·m^{-3}	**0.64**	0.082 m^3·m^{-3}	0.58
	Evaluation	**0.093** m^3·m^{-3}	**0.63**	0.081 m^3·m^{-3}	0.59

Table S3. Performances of different ET datasets (Unit of RMSD: mm·d^{-1}).

Dataset	Group	Overall RMSD	Overall CC	Median RMSD	Median CC
MOD16A2	Training	1.17 mm·d^{-1}	0.62	1.02 mm·d^{-1}	0.69
	Validation	1.04 mm·d^{-1}	0.68	1.03 mm·d^{-1}	0.75
	Evaluation	1.17 mm·d^{-1}	0.64	1.04 mm·d^{-1}	0.74
ETMonitor	Training	1.15 mm·d^{-1}	0.71	1.06 mm·d^{-1}	0.73
	Validation	1.13 mm·d^{-1}	0.72	1.05 mm·d^{-1}	0.75
	Evaluation	1.17 mm·d^{-1}	0.69	1.15 mm·d^{-1}	0.75
BERTH-30m	Training	0.69 mm·d^{-1}	**0.87**	0.61 mm·d^{-1}	0.84
	Validation	0.72 mm·d^{-1}	0.82	0.73 mm·d^{-1}	**0.84**
	Evaluation	**0.85 mm·d^{-1}**	**0.79**	0.77 mm·d^{-1}	0.83
BERTH-500m	Training	**0.65 mm·d^{-1}**	**0.87**	**0.60 mm·d^{-1}**	**0.85**
	Validation	**0.70 mm·d^{-1}**	**0.84**	**0.69 mm·d^{-1}**	0.83
	Evaluation	0.87 mm·d^{-1}	**0.79**	**0.76 mm·d^{-1}**	**0.84**

Table S4. Performances of BERTH (500m) over different land cover types.

Land Cover	Type	Overall MAD	Overall RMSD	Overall CC	Overall Bias
Forests	P	3.40 mm·d^{-1}	7.85 mm·d^{-1}	0.59	0.06 mm·d^{-1}
	SM	0.078 m^3·m^{-3}	0.100 m^3·m^{-3}	0.58	-0.025 m^3·m^{-3}
	ET	0.63 mm·d^{-1}	0.93 mm·d^{-1}	0.75	0.14 mm·d^{-1}
Shrublands	P	1.24 mm·d^{-1}	4.29 mm·d^{-1}	0.49	-0.01 mm·d^{-1}
	SM	0.043 m^3·m^{-3}	0.069 m^3·m^{-3}	0.79	0.025 m^3·m^{-3}
	ET	0.27 mm·d^{-1}	0.37 mm·d^{-1}	0.84	-0.01 mm·d^{-1}
Savannas	P	2.96 mm·d^{-1}	7.21 mm·d^{-1}	0.55	-0.05 mm·d^{-1}
	SM	0.085 m^3·m^{-3}	0.11 m^3·m^{-3}	0.65	-0.002 m^3·m^{-3}
	ET	0.53 mm·d^{-1}	0.78 mm·d^{-1}	0.80	0.02 mm·d^{-1}
Grasslands	P	2.05 mm·d^{-1}	5.69 mm·d^{-1}	0.51	-0.01 mm·d^{-1}
	SM	0.069 m^3·m^{-3}	0.087 m^3·m^{-3}	0.57	0.004 m^3·m^{-3}
	ET	0.57 mm·d^{-1}	0.81 mm·d^{-1}	0.81	-0.14 mm·d^{-1}
Croplands	P	2.42 mm·d^{-1}	6.08 mm·d^{-1}	0.52	-0.08 mm·d^{-1}
	SM	0.074 m^3·m^{-3}	0.094	0.63	0.018 m^3·m^{-3}
	ET	0.55 mm·d^{-1}	0.82 mm·d^{-1}	0.75	0.16 mm·d^{-1}

Table S5. Performances of BERTH (500m) when satellites overpass in situ stations or not.

Type	Time	Overall RMSD	Overall CC	Median RMSD	Median CC
P	Overpass	4.26 $mm \cdot d^{-1}$	0.46	3.55 $mm \cdot d^{-1}$	0.43
	Gap	8.13 $mm \cdot d^{-1}$	0.54	6.58 $mm \cdot d^{-1}$	0.51
SM	Overpass	0.088 $m^3 \cdot m^{-3}$	0.63	0.076 $m^3 \cdot m^{-3}$	0.58
	Gap	0.100 $m^3 \cdot m^{-3}$	0.59	0.084 $m^3 \cdot m^{-3}$	0.56
ET	Overpass	0.95 $mm \cdot d^{-1}$	0.80	0.78 $mm \cdot d^{-1}$	0.83
	Gap	0.80 $mm \cdot d^{-1}$	0.73	0.70 $mm \cdot d^{-1}$	0.79

Table S6. Performances of BERTH (30m) when satellites overpass in situ stations or not.

Type	Time	Overall RMSD	Overall CC	Median RMSD	Median CC
P	Overpass	4.44 $mm \cdot d^{-1}$	0.41	3.26 $mm \cdot d^{-1}$	0.41
	Gap	7.04 $mm \cdot d^{-1}$	0.52	5.46 $mm \cdot d^{-1}$	0.51
SM	Overpass	0.091 $m^3 \cdot m^{-3}$	0.62	0.073 $m^3 \cdot m^{-3}$	0.62
	Gap	0.094 $m^3 \cdot m^{-3}$	0.63	0.078 $m^3 \cdot m^{-3}$	0.58
ET	Overpass	0.79 $mm \cdot d^{-1}$	0.84	0.72 $mm \cdot d^{-1}$	0.86
	Gap	0.86 $mm \cdot d^{-1}$	0.78	0.77 $mm \cdot d^{-1}$	0.83

Table S7. All the data used in this study.

Name	Variables	Spatial Resolution	Temporal Resolution	Roles
Terra MODIS (MOD09GA)	Surface reflectance values at the red, green, blue, NIR, SWIR1, and SWIR2 bands	500 m	1 day	Model input
Aqua MODIS (MYD09GA)		500 m	1 day	
Landsat-7 ETM+		30 m	16 day	
Landsat-8 OLI		30 m	16 day	
Landsat-9 OLI2		30 m	16 day	
Sentinel-2 SLI		20 m	5 day	
ERA5-Land	Downwards solar radiation, downwards thermal radiation, surface pressure, 10-m u-component and v-component of wind, 2-m air temperature, and 2-m dewpoint temperature	0.1 degrees	1 hour	
Copernicus DEM GLO-30	Elevation	30 m	-	
MERIT	Elevation	90 m	-	
IMERG Final Run (V7B)	Precipitation	0.1 degrees	1 day	Pretraining
AMSR-E/2(MCCA)	Surface SM	0.1 degrees	1 day	
ETMonitor	ET	1 km	1 day	
GRFR	Runoff	0.05 degrees	1 day	
FLUXNET AmeriFLUX EuroFLUX	Latent heat flux	-	0.5 hour	Fine-tuning/ Evaluation
SC-EARTH	Precipitation	-	1 day	
ISMN	Surface SM	-	1 hour	
IMERG Early Run (V6)	Precipitation	0.1 degrees	1 day	Comparison
Enhanced SMAP (9 km)	Surface SM	9 km	1 day	
Enhanced SMAP (1 km)	Surface SM	1 km	1 day	
MOD16A2 (V6)	ET	500 m	8 day	
